# Autotitrating Bilevel Positive Airway Pressure in Large Vessel Steno-Occlusive Stroke Patients With Suspected Sleep Apnea: A Multicenter Randomized Controlled Study

**DOI:** 10.3389/fneur.2021.667494

**Published:** 2021-04-13

**Authors:** Kristian Barlinn, Stanislava Jakubicek, Timo Siepmann, Oleg Y. Chernyshev, Lars-Peder Pallesen, Miriam Wienecke, Wiebke Hermann, Xina Graehlert, Anne W. Alexandrov, Milan Vosko, Volker Puetz, Heinz Reichmann, Ulf Bodechtel, Robert Mikulik, Jessica Barlinn, Andrei V. Alexandrov

**Affiliations:** ^1^Department of Neurology, University Hospital Carl Gustav Carus, Technische Universität Dresden, Dresden, Germany; ^2^International Clinical Research Center and Neurology Department, St. Anne's University Hospital and Masaryk University, Brno, Czechia; ^3^Department of Neurology and Sleep Medicine, Louisiana State University Health-Shreveport, Shreveport, LA, United States; ^4^Departments of Neurology and Internal Medicine I, Interdisciplinary Sleep Centre, University Hospital Carl Gustav Carus, Technische Universität Dresden, Dresden, Germany; ^5^Department of Neurology, German Center for Neurodegenerative Diseases (DZNE), Rostock, Germany; ^6^Coordination Center for Clinical Studies, Technische Universität Dresden, Dresden, Germany; ^7^Department of Neurology, University of Tennessee Health Science Center, Memphis, TN, United States; ^8^Department of Neurology, Kepler University Hospital Linz, Linz, Austria

**Keywords:** acute ischemic stroke, sleep apnea, acute therapy, noninvasive ventilation, cerebral hemodynamics

## Abstract

**Background:** We hypothesized that autotitrating bilevel positive airway pressure (auto-BPAP) favorably affects short-term clinical outcomes in hyperacute ischemic stroke.

**Methods:** In a multicenter, randomized, controlled trial patients with large vessel steno-occlusive stroke and clinically suspected sleep apnea were allocated to auto-BPAP or standard stroke care alone. Auto-BPAP was initiated within 24 h from stroke onset and performed over 48 h during diurnal and nocturnal sleep. Sleep apnea was assessed using cardiorespiratory polygraphy. Primary endpoint was early neurological improvement on National Institutes of Health Stroke Scale (NIHSS) score at 72 h. Safety and tolerability of BPAP, functional independence [modified Rankin Scale (mRS) 0–2], stroke recurrence, and mortality at 90 days were assessed.

**Results:** Due to low recruitment, the trial was prematurely stopped after 24 patients had been randomized (auto-BPAP, *n* = 14; control, *n* = 10): median baseline NIHSS 13 (5.5–18), 88% large vessel occlusion, and 12% large vessel stenosis. Polygraphy confirmed sleep apnea in 64% of auto-BPAP and 88% of control patients (*p* = 0.34). Adherence to auto-BPAP was achieved by 9 of the 14 (64%) patients. Between auto-BPAP and control patients, no differences were observed in early neurological improvement (median NIHSS change: −2.0, IQR = 7 points vs. −0.5, IQR = 3 points), 90 days functional independence (21 vs. 30%, *p* = 0.67), stroke recurrence (0 vs. 20%, *p* = 0.16), and death (14 vs. 20%, *p* = 1.0). No safety concerns were identified.

**Conclusions:** In this prematurely terminated trial, auto-BPAP was safe but did not show an effect on short-term clinical outcomes in selected ischemic stroke patients. Its tolerability, however, may be limited in hyperacute stroke care and needs to be improved before larger trials are conducted.

**Clinical Trial Registration:** ClinicalTrials.gov, identifier: NCT01812993.

## Introduction

Approximately 70% of stroke patients suffer from sleep apnea, and its presence is associated with early neurological worsening, adverse clinical outcomes, and increased mortality (1). Noninvasive ventilation with continuous positive airway pressure (CPAP) was shown to reduce long-term mortality and risk of recurrence in ischemic stroke patients with sleep apnea (2, 3). Although most studies were conducted in the subacute or rehabilitation phases of stroke, greatest neurological improvement is likely to be achieved among patients who start noninvasive ventilation within 48 h from stroke onset (4).

We recently hypothesized that intermittent hypercapnia in ischemic stroke patients with sleep apnea may further compromise perfusion in potentially salvageable brain tissue when vasodilation in the nonaffected brain region leads to blood flow diversion from ischemic area to the nonischemic regions (1). This cerebral blood flow steal phenomenon was shown to be particularly evident in ischemic stroke patients with large vessel occlusion and excessive daytime sleepiness, and it was associated with a four-fold risk of neurological deterioration, possibly the clinical surrogate for penumbra progression of infarction (5, 6). Moreover, our recent observational data showed that these patients were more likely to improve neurologically during hospital course when bilevel positive airway pressure (BPAP) was started within first 24 h of admission than those who did not receive BPAP (7). Other studies on early noninvasive ventilation in acute stroke, however, predominantly recruited patients with heterogeneous stroke etiologies and did not report frequency of causative steno-occlusive lesions (8).

The primary objective of this study was to test the hypothesis that patients with large vessel steno-occlusive stroke and suspected sleep apnea may benefit from noninvasive ventilation with auto-BPAP initiated within 24 h of symptom onset.

## Methods

This multicenter, randomized, controlled, parallel-group phase II trial, the Reversal of the Neurological Deficit in Acute Stroke With the Signal of Efficacy Trial of Auto BPAP to Limit Damage From Suspected Sleep Apnea (Reverse-STEAL) trial, was conducted in compliance with the consolidated standards of reporting trials (CONSORT) statement (9). The complete trial protocol has been published previously (10). Briefly, consecutive patients aged 18–80 years presenting to the participating centers (Dresden, Germany; Brno, Czech Republic, Memphis, USA; Shreveport, USA; Linz, Austria) were eligible for the study if the following inclusion criteria were met: (1) ischemic stroke with measurable deficit [National Institutes of Health Stroke Scale (NIHSS) score ≥4 points] within 24 h from symptom onset; (11) causative extracranial (i.e., internal carotid artery) or intracranial (i.e., internal carotid artery; middle or anterior or posterior cerebral arteries) ≥50% stenosis, near-occlusion or occlusion; and (2) high-risk of having sleep apnea [defined by Berlin questionnaire (12)], history of known sleep apnea, or witnessed repetitive apnea episodes during sleep or somnolence during hospitalization. Patients with vertebrobasilar stroke, known sleep apnea currently on noninvasive ventilation, and premorbid modified Rankin scale (mRS) score ≥3 were excluded from the study.

Patients were allocated to standard stroke care alone without any ventilatory treatment or standard stroke care plus auto-BPAP in stratified randomization blocks with strata for study site and stroke severity as defined by the NIHSS. Auto-BPAP (Philips Respironics, Auto-BIPAP Biflex®, Germany) applied by individually adjusted full-face mask was initiated within 24 h from symptom onset and maintained for a maximum of 48 h during diurnal and nocturnal sleep or somnolence. The auto-BPAP device automatically adjusted expiratory (EPAP) and inspiratory positive airway pressure (IPAP) levels to meet patients' needs sparing manual titration of therapeutic pressures. Pressure settings were identical in all patients in the active group: minimum EPAP, 4 mbar; possible maximum IPAP, 25 mbar; maximum difference between EPAP and IPAP (delta), 8 mbar; with moderate expiratory breathing relief function (Biflex level 2). Between days 3 and 5 from enrollment, all patients underwent diagnostic cardiorespiratory polygraphy for assessment of sleep apnea. Polygraphy comprised recording of oronasal airflow, microphone, thoracic and abdominal respiratory effort, body position, and oxygen saturation. Polygraphic recordings were scored by an experienced sleep medicine specialist blinded to group allocation. Scoring of respiratory events followed standard recommendations of the American Academy for Sleep Medicine (AASM) (13). Single respiratory events were classified as hypopnea (decrease of SpO_2_ saturation ≥3% and reduction of flow ≥30%) or apneas (reduction of flow ≥90%). Apneas were classified as obstructive, central, or mixed according to AASM criteria. Sleep apnea was defined as the sum of hypopneas and apneas per hour with a minimum Apnea–Hypopnea Index (AHI) >5/h on polygraphy. Sleep apnea severity was classified according to established definitions as mild (AHI > 5/h < 15/h), moderate (AHI ≥ 15 < 30/h), or severe (AHI ≥ 30/h).

The primary endpoint was any early neurological improvement on NIHSS score at 72 ± 12 h from randomization. Secondary endpoints comprised adherence to auto-BPAP (defined as tolerating treatment during sleep or somnolence for at least 4 h continuously) and safety including (1) serious adverse events during treatment period (for a maximum of 72 h from treatment initiation) related to auto-BPAP, (11) death during hospital stay, and (2) all complaints and possible side effects of auto-BPAP. We also assessed (1) neurological worsening (increase in baseline NIHSS score ≥4 points) at 24, 48, and 72 h from randomization; (11) early neurological improvement (decrease in baseline NIHSS score ≥ 4 points) at 24, 48, and 72 h from randomization; (2) favorable functional outcome (mRS score 0–2) at discharge and at 90 days; and (3) any transient ischemic attack (TIA) or new ischemic stroke during hospitalization or within 90 days of protocol initiation. All efficacy endpoints were obtained by investigators blinded to group allocation.

The research protocol was approved by the institutional review boards of each participating study center, and all patients and/or their legal authorized representatives provided informed consent. This trial was registered on ClinicalTrials.gov (NCT01812993).

### Statistical Analysis

Data of intention-to-treat population were analyzed. For confirmatory analysis, the primary endpoint was assessed using Mann–Whitney *U*-test. For secondary endpoints, categorical variables were assessed using chi-square tests or Fisher's exact test, while continuous variables were assessed using Student's t test and Wilcoxon rank sum test, where appropriate. Complete case analysis was used to handle missing data. Statistical significance was set as *p* < 0.05. All statistical analyses were performed with STATA software (Version 12.1, StataCorp., College Station, TX).

## Results

Due to low patient accrual, the trial was stopped early in July 2017 after 25 of 60 planned patients [75% male; mean age, 67.4 ± 8.5 years; median baseline NIHSS score, 12.5 (IQR 5.5–18) points; 88% large vessel occlusion] were randomized over a study period of 43 months. One of these patients was excluded from the study prior to initiation of any study specific procedures due to large hemispheric infarction and severe dysphagia. In total, 24 patients were included in this analysis (auto-BPAP, *n* = 14; control, *n* = 10). A detailed description of demographic values, comorbidities, and clinical and radiographic data is shown in Table 1.

**Table 1:** Patient characteristics.

**Variable**	**Auto-BPAP (*n* = 14)**	**Control (*n* = 10)**	***p***
Age, mean ± SD	65.1 ± 8.5	70.6 ± 7.8	0.12
Men, *n* (%)	9 (64.3)	9 (90)	0.34
Body mass index, mean ± SD	28.4 ± 5.1	25.8 ± 3.7	0.19
History of risk factors, *n* (%)			
Arterial hypertension	14 (100)	9 (90)	0.42
Dyslipidemia	5 (35.7)	4 (40)	1.0
Diabetes mellitus	5 (35.7)	4 (40)	1.0
Tobacco use	2 (14.3)	3 (30)	0.62
Coronary heart disease	3 (21.4)	3 (30)	0.67
Atrial fibrillation	3 (21.3)	2 (20)	1.0
Congestive heart failure	2 (14.3)	1 (9)	1.0
Sleep apnea	0 (0)	1 (9)	0.42
Baseline NIHSS score, median (IQR)	12.5 (7–18)	12.5 (4–18)	0.72
Intracranial vessel occlusion, *n* (%)	13 (92.9)	8 (80)	0.55
Middle cerebral artery	11 (78.6)	8 (80)	1.0
Terminal internal carotid artery/carotid-T	3 (21.4)	3 (30)	0.67
Anterior cerebral artery	1 (7.1)	0	1.0
Posterior cerebral artery	2 (14.3)	0	0.49
Symptomatic ≥70% carotid stenosis, *n* (%)	3 (21.4)	3 (33.3)	0.64
Extracranial carotid occlusion, *n* (%)	3 (21.4)	3 (30)	0.67
Intravenous thrombolysis, *n* (%)	7 (50)	3 (30)	0.42
Endovascular therapy, *n* (%)	0 (0)	0 (0)	−

*SD, standard deviation; NIHSS, National Institutes of Health Stroke Scale; IQR, interquartile range*.

Adherence to auto-BPAP was given in 9 of 14 (64%) patients. Four of 14 (29%) patients refused continuation of noninvasive ventilation due to intolerance or local complaints (i.e., local irritation of skin/mucosa and mucosal dryness). In one patient (7%), continuous treatment with auto-BPAP was not possible due to severe agitation. Median baseline NIHSS score of these patients was 9 (IQR = 6–19) points. The average time of auto-BPAP used in all patients was 13.17 ± 11.08 h. Mean usage time of auto-BPAP was lower in patients who did not tolerate treatment than in patients who adhered to treatment (2.26 ± 2.20 vs. 19.22 ± 9.02 h, *p* = 0.002).

While one patient in the control group suffered from aspiration pneumonia, no patient in the intervention group experienced any respiratory complications during the treatment period (10 vs. 0%, *p* = 0.42). One patient in the active group died from acute heart failure following myocardial infarction at the 2nd day of treatment initiation, which was not considered causatively related to BPAP ventilation comprising 5.4 h in total. Neurological worsening within 72 h from BPAP initiation was related to progression of infarction in three patients, middle cerebral artery reocclusion in one patient, and reinfarction in another patient.

No difference was observed in the primary endpoint of any neurological improvement at 72 h between auto-BPAP (median NIHSS change: −2.0, IQR = −3 to 4 points) and control (median NIHSS change: −0.5, IQR = −2 to 1 points) groups (*p* = 0.69). This finding remained unchanged when we excluded the five patients with nonadherence to auto-BPAP from the analysis (*p* = 0.87). Further secondary neurological outcomes are detailed in Table 2.

**Table 2:** Adverse events and secondary clinical outcomes.

**Variable**	**Auto-BPAP (*n* = 14)**	**Control (*n* = 10)**	***p***
Adverse events, *n* (%)			
Aspiration pneumonia	0	1 (9)	0.42
Myocardial infarction	1 (7.1)	0	1.0
Stroke progression	3 (21.4)	0	0.24
Middle cerebral artery reocclusion	1 (7.1)	0	1.0
Reinfarction in remote vascular territory	0	1 (9)	0.42
Early neurological improvement, *n* (%)			
24 h	0 (0)	0 (0)	−
48 h	3 (21.4)	1 (9)	0.6
72 h	3 (21.4)	2 (20)	1.0
Early neurological deterioration, *n* (%)			
24 h	1 (7.1)	0 (0)	1.0
48 h	3 (21.4)	0 (0)	0.2
72 h	4 (30.8)^†^	1 (9)	0.3
Favorable functional outcome 90 days, *n* (%)	3 (21.4)	3 (30)	0.7
Death 90 days, *n* (%)	2 (14.3)	2 (20)	1.0
Recurrent stroke 90 days, *n* (%)	0 (0)	2 (20)	0.2

† *According to 13 patients (one patient was deceased at the time of follow-up)*.

Cardiorespiratory polygraphy could not be performed in 4 of 24 (17%) patients because of clinical deterioration. The polygraphic recording was not analyzable in 1 of 24 (4%) patients due to insufficient quality of recordings. Sleep apnea was confirmed in 7 of 11 patients (64%) of auto-BPAP and 7 of 8 (88%) of control patients.

To assess whether false positive clinical suspicions of sleep apnea might have skewed our observations, we repeated our analysis considering only those patients with confirmation of sleep apnea on polygraphy. However, this subgroup also showed no difference in the primary endpoint between auto-BPAP (median NIHSS change: −1.0, IQR = −3 to 4 points) and control (median NIHSS change: 0, IQR = −2 to 1 points; *p* = 0.75). The results of the polygraphy are summarized in Table 3.

**Table 3:** Results of cardiorespiratory polygraphy.

**Variable**	**Auto-BPAP (*n* = 11)**	**Control (*n* = 8)**	***P***
Apnea–hypopnea index, median (IQR)	7.2 (1.2–17)	13.2 (8.9–26.1)	0.16
Sleep apnea, *n* (%)	7 (63.6)	7 (87.5)	0.34
Obstructive sleep apnea	7 (63.6)	6 (75.5)	
Central sleep apnea	0 (0)	0 (0)	
Mixed sleep apnea	0 (0)	1 (12.5)	
Sleep apnea severity, *n* (%)			0.8
Mild	4 (36.4)	3 (37.5)	
Moderate	2 (18.2)	3 (37.5)	
Severe	1 (9.1)	1 (12.5)	

*IQR, interquartile range*.

## Discussion

The major finding of our study is that noninvasive ventilation with auto-BPAP is safe but may not be feasible in the setting of hyperacute stroke, as demonstrated by limited adherence to treatment in the setting of a randomized controlled multicenter study.

This might be explained by several reasons. First, although autotitrating BPAP is perceived as more comfortable in the general population than CPAP ventilation, its tolerance in one-third of our specific stroke population was possibly altered by stroke-related neurological deficits hindering appropriate understanding and compliance to allow patients to tolerate noninvasive ventilation by facial masks. In fact, individual discomfort and agitation were the most common reasons for BPAP intolerance in our patients with NIHSS scores, indicative of moderate to severe stroke severity. These observations are in line with a recent randomized study that deployed auto-CPAP ventilation in acute stroke patients within 48 h from symptom onset (14). Adherence to CPAP was 62.5% in 16 patients who were advised to use auto-CPAP for 30 days. No data on CPAP adherence was reported for the first 48 h in this study. Another randomized study utilized auto-CPAP in the first three nights after stroke (8). Only 40% of 25 stroke patients tolerated constant noninvasive ventilation for at least 4 h per night, with higher neurological disability being a negative predictor of CPAP adherence. Second, the trial was conducted in centers experienced with sleep medicine, yet nightly supervision of BPAP therapy was predominantly provided by regular medical staff that generally does not have in-depth expertise to counteract patient difficulties with noninvasive ventilatory treatment. Lastly, the evolving role of endovascular therapy and the extension of therapeutic time windows in ischemic stroke in recent years competitively slowed down recruitment of patients with persistent large steno-occlusive stroke into this trial. The fact that none of our patients underwent endovascular therapy supports our assumption that competitive treatment strategies could have been an important reason for inclusion bias.

Even though neurological improvement appeared slightly higher in the auto-BPAP group than in the control group (−2 vs. −0.5 points on the NIHSS score), our trial does not reach adequate power to confirm or reject potentially beneficial effects of hyperacute treatment with auto-BPAP in selected ischemic stroke patients with large vessel disease and sleep apnea. However, the average time patients were exposed to auto-BPAP was 13 h, a duration far beyond that one postulates as minimum to expect any positive effects on vascular outcomes in a general population with sleep apnea (15). Moreover, results of cardiorespiratory polygraphy confirming our previous findings of high frequency of sleep apnea in hyperacute stroke patients underline internal validity of our trial (16). We therefore speculate that a positive effect on neurological recovery, if any, could have been detected in patients tolerating the mask sufficiently given that the estimated sample size was reached. This hypothesis is supported by recent randomized data showing greater early neurological improvement in acute ischemic stroke patients with CPAP adherence for more than 4 h compared with those whose adherence was shorter (−2.3 vs. −1.4 points on the NIHSS score) (8). Patients in this study, however, were treated with auto-CPAP for longer time periods and suffered from more severe sleep apnea as shown by higher AHI scores. Moreover, Bravata et al. (14) found that greater improvement in the NIHSS score was observed in ischemic stroke patients with sleep apnea with increasing auto-CPAP use (−3.0 points for patients with good auto-CPAP adherence vs. −1.0 points for control patients). Application of more extended BPAP treatment might have led to a more favorable outcome also in our patients.

Application of auto-BPAP appeared safe in our trial. Patients exposed to noninvasive ventilation did not experience medical complications that were causatively related to the intervention. Most notably, severe aspiration or respiratory failure was absent in all treated patients, a common concern in ischemic stroke patients frequently suffering from dysphagia who require noninvasive ventilation for any reason.

From a pathophysiological perspective, potential harmful effects of sleep apnea in patients with ischemic stroke include intermittent hypoxemia, cardiac arrhythmias, and rapid blood pressure fluctuations (1). Ischemic brain tissue particularly in the setting of large steno-occlusive disease already depends on dilated arteries with no further or only minimal residual vasomotor capacity to counteract apnea-associated blood pressure changes and to maintain blood flow according to local metabolic needs. In addition, hypercapnia may lead to depletion of the collateral blood flow due to blood flow diversion from the ischemic area to the nonischemic areas along the path of least resistance (1). The novelty of this trial was the inclusion of selected patients with large vessel steno-occlusive stroke, since their brain resistance arteries are already set to maximum dilation with no or only minor blood flow increase possible in response to vasodilatory stimuli. In contrast, other studies primarily targeted at unselected stroke patients without considering stroke etiologies (8, 14). In addition, the utilization of an autotitrating BPAP device within the first 24 h of stroke without the need of preceding time-consuming sleep diagnostics and manual titration of the ventilation device constituted another novel aspect of this trial.

Our research protocol did not provide monitoring of cerebral hemodynamics and blood gases. Therefore, to what extent noninvasive ventilation eventually has reversed potentially deleterious effects of sleep apnea on cerebral blood flow remains uncertain in our trial. Lastly, we did not assess nursing workload among both groups. Therefore, we can only speculate whether increased nursing time would have improved patients' adherence to BPAP therapy.

In summary, we were not able to confirm or refute the hypothesized beneficial effects of auto-BPAP ventilation on short-term clinical outcomes following ischemic stroke due to limited compliance with BPAP. Auto-BPAP therapy, however, did not have any serious adverse effects. There is still a need for well-tailored clinical trials of sleep apnea treatment in patients with acute ischemic stroke given its burden of vascular morbidity and mortality. Building up a lesson from our prematurely stopped trial, auto-BPAP may still be an alternative ventilation mode to be further investigated in the hyperacute stroke setting, provided that its utilization is covered by round-the-clock, well-trained personnel to ensure patients' adherence and properly determine its intervention effects.

## Data Availability Statement

The raw data supporting the conclusions of this article will be made available by the authors, without undue reservation.

## Ethics Statement

The studies involving human participants were reviewed and approved by Institutional Review Board of the Technische Universitaet Dresden. The patients/participants provided their written informed consent to participate in this study.

## Author Contributions

KB: conception of study, acquisition, analysis and interpretation of data, drafting the manuscript, and final approval. SJ, MV, and UB: conception of study, acquisition of data, revising the manuscript, and final approval. TS, OC, L-PP, and MW: acquisition of data, revising the manuscript, and final approval. WH: acquisition and analysis of data, revising the manuscript, and final approval. XG, AWA, and HR: conception of study, analysis of data, revising the manuscript, and final approval. VP, RM, JB, and AVA: conception of study, acquisition and analysis of data, revising the manuscript, and final approval. All authors contributed to the article and approved the submitted version.

## Conflict of Interest

The authors declare that the research was conducted in the absence of any commercial or financial relationships that could be construed as a potential conflict of interest.
